# Risk Factors, Associations, and Outcomes of Reduced Fetal Movements: A Preliminary Cross-Sectional Study at Port Sudan Maternity Hospital

**DOI:** 10.7759/cureus.73628

**Published:** 2024-11-13

**Authors:** Mohammed Ali Saad Ali, Maysa Azhari Hamid Mohammed, Hadeel Mohamed Muaid Abdelseid, Eithar M Ali

**Affiliations:** 1 Obstetrics and Gynecology, Red Sea University, Port Sudan, SDN; 2 Obstetrics and Gynecology, Red Sea State Ministry of Health, Port Sudan, SDN; 3 Medicine, University of Gadarif, Qadarif, SDN; 4 Medicine, University of Khartoum, Khartoum, SDN

**Keywords:** antenatal care, fetal movement, perinatal mortality, reduced fetal movement, stillbirth

## Abstract

Background: Reduced fetal movements (RFM) are a significant concern in obstetric care. They often indicate fetal distress and are associated with adverse perinatal outcomes such as stillbirth and intrauterine growth restriction (IUGR). While RFM is recognized as a critical marker of fetal well-being, there is a limit to the data available on its risk factors and outcomes in the Port Sudan region. This study aimed to estimate risk factors and outcomes of pregnancies at risk due to RFM.

Methods: This was a cross-sectional hospital-based study, conducted from February to August 2022 at Port Sudan Maternity Hospital, focused on mothers with RFMs defined by the RCOG guidelines. A total of 33 cases were analyzed using a structured questionnaire, with data collected via direct interviews. Data analysis was performed using SPSS version 26.0 (IBM Corp., Armonk, NY), employing descriptive statistics, frequency tables, and cross-tabulations. Ethical approval was obtained, and participant confidentiality was ensured through anonymization.

Results: Most participants were married women aged 18 to 29 years, primarily housewives with diverse educational backgrounds. A minority reported medical conditions like diabetes (6.1%) and hypertension (3%), while pregnancy complications included preeclampsia (10%) and anemia (6.7%). The first episode of RFM was commonly reported before 28 weeks of gestation (34.4%). Normal vaginal delivery was the most frequent mode of birth (48.4%), with a significant number of pregnancies resulting in intrauterine fetal demise (IUFD) (53.3%). Additionally, 77.8% of newborns had five-minute APGAR scores below 7, and 42.9% had birth weights between 2.5 kg and 3.5 kg. About 12.1% of births in our study were stillbirths and all of them were preterm babies.

Conclusion: The study underscores the frequent serious implications of RFM on pregnancy outcomes with the majority presenting as low APGAR scores and IUFD. Early detection and timely management of RFM are crucial for improving maternal and neonatal outcomes. Tailored antenatal care (ANC) is needed to address the diverse risks associated with RFM.

## Introduction

Maternal perception of fetal movement is an early sign of fetal life, often indicating fetal well-being. Typically, mothers begin to notice these movements, such as kicks, flutters, or rolls, between 18 and 20 weeks of pregnancy. These movements soon follow a regular pattern, and any notable decrease or abrupt change in this activity is defined as reduced fetal movement (RFM) and can be a significant clinical indicator [1]. It is a frequently seen problem in maternity care, with 6-15% of women having at least one episode of RFM throughout the third trimester of gestation [2]. The frequency of spontaneous movements increases until the 32nd week of pregnancy, then plateaus until the onset of labor. The type of fetal movement may change in the third trimester but there is no reduction in the frequency. By term, the number of movements per hour ranges from 16 to 45 and the longest period is between 50 and 75 minutes. From 20 weeks, fetal movements show diurnal changes with afternoon and evening peaks. Fetal sleep cycles are usually between 20 and 40 minutes and it often does not exceed 90 minutes in a normal fetus [3].

RFM, which is defined as a personal observation of significantly decreased or absent fetal activity, has been developing as a key clinical indicator to identify women at high risk of stillbirth and intrauterine fetal death due to placental dysfunction [4]. In this instance, the majority of stillbirths seem to be preceded by a period of RFM lasting three to four days, and RFMs also occur before fetal death in 55% of women who have experienced stillbirths [5-8]. Recently, it has been shown that women with RFMs have irregular placental morphology and function, signifying a possible association between placental insufficiency and the presence of RFMs [9,10]. Several recent studies have reported that uterine artery (UtA) Doppler indices and pregnancy-associated plasma protein (PAPP)-A are both related to poor trophoblastic development and to placenta-related complications such as preeclampsia and intrauterine growth restriction (IUGR) [11-18]. Additionally, the stillbirth rate is an important factor in evaluating maternal health and the quality of maternity care during pregnancy and birth. Throughout the previous decade, there have been calls for further reduction in stillbirth rates [19]. Preventing and reducing adverse outcomes can only be achieved through better detection and management of women with RFM. However, the characteristics of women who present with RFM in pregnancy vary, and risk factors for RFM are increasingly being explored with numerous associated features, including maternal age, BMI, parity, ethnicity, smoking, occupation, the position of the placenta, and medications, reported across studies. Studies that have been conducted on fetal movements in pregnancy to date have focused on methods of fetal movement counting to assess fetal well-being, management of reported RFM during pregnancy, and interventions to improve maternal awareness of RFM [20-22].

Risk factors associated with women who present with RFMs as a primary complaint during pregnancy were identified. Women experiencing RFMs were more likely to be Caucasian and have conditions such as an anterior placenta, oligohydramnios, or polyhydramnios (non-modifiable factors), with smoking being a modifiable factor [23]. No significant differences were observed in parity or between the average characteristics of women with RFM and those without. Additionally, factors such as a history of cesarean sections, pregnancies extending beyond 42 weeks, and medical conditions like diabetes and hypertensive disorders were not found to be predictive of RFM during pregnancy [23]. There is a proven record of increasing the burden of care required by women with RFM, including increased neonatal unit admission rates, increased induction rates, and higher surveillance demands, demonstrating the need for increased attention to this area of practice [24]. Repeated episodes of RFM have been linked to increased risk of adverse outcomes such as perinatal mortality, neonatal unit admission, abnormal cardiographs at presentation, a composite severe morbidity outcome, birth weight, induction of labor, cesarean section, admission rates, and ultrasound usage rates [25]. In general, early identification by healthcare professionals of risk factors, both modifiable and non-modifiable, that are significantly associated with RFM could contribute to the prevention and reduction of adverse pregnancy, birth, fetal, and neonatal outcomes.

The study was conducted to identify maternal and fetal risk factors for RFM in pregnancy and to assess pregnancy, labor, and birth outcomes following RFM. 

## Materials and methods

Study setting, population, and sampling

This hospital-based cross-sectional observational study was conducted over six months from February to August 2022 at the Department of Obstetrics and Gynecology, Port Sudan Maternity Hospital, a government-run facility situated in Port Sudan City. The hospital offers tertiary care services for women receiving antenatal care (ANC), providing round-the-clock obstetrics and gynecology services, including a labor room and outpatient clinic. It caters to patients from Port Sudan City and its neighboring areas and referrals from other medical facilities. Women with risk factors or obstetric complications are specifically directed to this hospital. The labor room is equipped with 10 beds and six delivery tables, and the facility also houses a daily referred clinic, elective major and minor obstetrics and gynecology operating theaters, inpatient wards with 40 beds, and an Intensive Care Unit with six beds. The hospital serves patients from the Red Sea State, encompassing Port Sudan City and its environs.

The study population included pregnant women presenting with RFM at or beyond 28 weeks of gestation. Women with singleton pregnancies who had experienced RFM at least once and were able to provide informed consent were included. Exclusion criteria included known fetal abnormalities, multiple pregnancies, or communication barriers due to the wide variety of local dialects spoken by the tribal groups in the Red Sea state, which sometimes posed challenges in ensuring clear understanding in the absence of a local translator.

The sample size was calculated according to the equation of an unknown size population: n = Z² x P x (1-P) \ E². Where n is the sample size, Z²: square of normal standard deviation = 3.8416, P is the probability of occurrence = 0.05, (1-P) is the probability of non-occurrence = 1-0.05 = 0.95, and E²: square of margin of error = 0.0025. Ended with an estimate of 72, but 33 cases were included due to time constraints. A comprehensive sampling technique was applied involving total coverage of cases across labor rooms, emergency departments, and elective units.

Data collection

Data was collected using a structured, closed-ended questionnaire divided into four subsections: socio-demographic data: capturing the basic demographic details of the participants; maternal factors, including parity, weight, smoking status, timing of ANC, medical illnesses, quickening (first fetal movement), and pregnancy complications; fetal and gestational factors: addressing gestational age at the onset of RFM, fetal weight, amount of glucose intake during reduced movement episodes, and whether the fetus had an anterior placenta; the last section is about the delivery outcomes, including the mode and place of delivery, the outcome, and the gestational age at the time of delivery. Direct face-to-face interviews were conducted to ensure accurate data collection.

Data entry and analysis were carried out using SPSS version 26.0 (IBM Corp., Armonk, NY). Descriptive statistics were generated in the form of frequency tables and percentages for categorical variables and means with standard deviations for continuous data. Inferential statistics were applied to examine relationships between key variables, with a P-value ≤ 0.05 considered significant. The results were presented through univariate and bivariate tables, graphs, and narrative descriptions for clarity.

Ethical consideration and approval

Ethical approval was granted by the Red Sea’s Ministry of Health Ethical Committee and the Sudan Medical Specialization Board (SMSB), Council of Obstetrics and Gynecology. Permissions from the hospital management were also obtained from the general manager and the medical director. Written informed consent was secured from all participants, and confidentiality and privacy were maintained by anonymizing the data using serial numbers. The data were exclusively used for research purposes in compliance with ethical standards.

## Results

Socio-demographic characteristics

The study participants from Port Sudan displayed a diverse demographic profile. Nearly half of the participants (48.5%) were between 18 and 29 years old. A large majority were married (93.9%), with 66.7% identifying as housewives. Educational levels varied, with 30.3% having completed primary school and 21.2% having attended college. Additionally, 32.3% of the women were married between the ages of 13 and 17 (Table 1).

**Table 1:** Socio-demographic characteristics of the participants from Port Sudan

Variable	Category	N	%
Age	Less than 18 years	4	%12.1
	18 - 29 years	16	48%
	30 - 45 years	13	39.4%
Marital status	Married	31	93.9%
	Divorced	1	3.0%
	Widowed	1	3.0%
Job	Unemployed	3	9.1%
	Doctor	2	6.1%
	Farmer	2	6.1%
	Housewife	22	66.7%
	Teacher	4	12.1%
Education	None	8	24.2%
	Khalwa (religious retreat)	2	6.1%
	Primary school	10	30.3%
	Secondary school	5	15.2%
	College	7	21.2%
	Graduate	1	3.0%
Age of marriage	Not sure	1	3.2%
	Less than 13 years	7	22.6%
	13 - 17 years	10	32.3%
	18 - 25 years	8	25.8%
	26 - 33 years	7	16.1%

Maternal factors for RFM

In terms of parity, the majority of participants (42.4%) were primagravida, while only 24.2% had more than five pregnancies. For BMI, 54.5% of participants had a BMI between 18 and 25. Regarding smoking, 96.9% of participants had never smoked, 3.1% were current smokers, and none had a history of past smoking. As for ANC, 68.8% of participants had regular ANC visits, and 31.3% had none. Regarding the reasons for not being on regular and treated care, respondents provided the same responses (12.5%) for the following options: could not come to the hospital, financial issues, habits, irregular or rural areas, not clear reasons, residents far from the hospital and not dedicated enough for ANC, she thinks it does not need follow-up and the baby will be fine, and the respondent was on contraceptives and was not aware of the pregnancy (Table 2). Concerning quickening (first fetal movement), 43.8% of participants reported noticing movement before 18 weeks, while 3.1% did not appreciate fetal movement. When it came to medical conditions, 90.6% of participants had no medical illnesses, 3.1% had hypothyroidism, 3.1% had hypertension, and 6.3% had diabetes. Finally, 56.7% of participants reported no pregnancy complications (Table 2).

**Table 2:** Maternal factors for RFM *Multiple answers were allowed. ANC, antenatal care; PROM, premature rupture of membranes; APH, antepartum hemorrhage; PPH, postpartum hemorrhage; DM2, type 2 diabetes mellitus

Categories		Answers	
		N (n = 33)	%
Parity	Primigravida	14	42.4%
	G1-5	11	33.3%
	More than 5	8	24.2%
BMI		N (n = 33)	%
	Less than 18	3	9.1%
	18-25	18	54.5%
	More than 25	8	24.2%
	Not assessed	4	21.1%
History of smoking		N (n = 33)	%
	No history of smoking	32	96.9%
	Current smoker	1	3.1%
	Previous smoker	0	0%
ANC visits		N (n = 33)	%
	No	8	31.3%
	Yes	25	68.8%
Reasons for not being on regular ANC		N (n = 8)	%
	Couldn’t come to hospital	1	12.5%
	Financial issues	1	12.5%
	Habits	1	12.5%
	Irregular, rural area	1	12.5%
	No clear reasons	1	12.5%
	Residence far from hospital and not educated enough for ANC	1	12.5%
	She thinks it doesn't need follow-up, she and the baby will be fine	1	12.5%
	The patient was on contraceptive pills and wasn't aware of the pregnancy	1	12.5%
Quickening (first fetal movement)		N (n = 33)	%
	Didn't appreciate fetal movement	2	3.1%
	Before 18th week	14	43.8%
	19th to 22nd week	12	37.5%
	After 22nd week	5	15.6%
Medical illness*		N (n = 33)	%
	DM	2	6.3%
	HTN	1	3.1%
	Hypothyroidism	1	3.1%
	None	29	90.6%
Pregnancy complications*		N (n = 33)	%
	Obstructed labor	1	3.3%
	APH	1	3.3%
	Ruptured uterus	1	3.3%
	PIH	1	3.3%
	GDM	1	3.3%
	PROM	2	6.7%
	Eclampsia	2	6.7%
	PPH	2	6.7%
	Anemia	2	6.7%
	Infection	2	6.7%
	Pre-eclampsia	3	7.9%
	DM2	3	7.9%
	None	17	56.7%

Fetal risk factors for RFM

The evaluation of fetal risk factors related to RFM showed that the majority of participants experienced their initial episode of RFM at 28 weeks of gestation (34.4%). Regarding amniotic fluid levels at the time of the first RFM episode, the majority had average levels (56.3%), and 56.2% of respondents were not sure about the position of the placenta at the first episode of RFM (Table 3). 

**Table 3:** Fetal risk factors for RFM RFM, reduced fetal movements

Categories		Answers	
		N (n = 33)	%
Gestational age at first episode of RFM	28-30	12	34.4%
	31-32	7	21.9%
	33-34	6	18.8%
	35 or more	8	25.0%
Estimated fetal weight at the time of first episode of RFM		N (n = 33)	%
	650-1300 g	3	9.1%
	1400-1900 g	18	54.5%
	2000-2400 g	0	%0.0
	2500 g or more	8	24.2%
	Not sure	4	12.1%
Amount of liquor at the time of first episode of RFM		N (n = 33)	%
	Oligohydramnios	12	37.5%
	Average	18	56.3%
	Polyhydramnios	3	6.3%
Did you have anterior placenta at the time of 1stepisode of RFM		N (n = 32)	%
	Yes	11	31.2%
	No	9	28.1%
	Not sure	13	56.2%

Delivery and pregnancy outcomes

Delivery data indicated that the most common mode of delivery was normal vaginal delivery (48.4%), followed by emergency cesarean section (32.3%). The majority of births occurred in hospital settings (83.8%). Gestational age at delivery varied, with a notable portion between 37 and 40 weeks (46.7%). Pregnancy outcomes related to RFM included intrauterine fetal demise (IUFD) (53.3%), live births (23.3%), and stillbirths (13.3%). The five-minute APGAR scores showed that most newborns had scores below 7 (77.8%). Regarding birth weight, a substantial portion of infants weighed between 2.5 kg and 3.5 kg (42.9%). Overall, the study offered valuable insights into the demographic details, medical history, pregnancy complications, fetal risk factors, delivery date, and pregnancy outcomes of participants from Port Sudan (Table 4).

**Table 4:** Delivery and pregnancy outcomes

Categories		Answers	
		N (n = 31)	%
Mode of delivery	Normal vaginal delivery	16	48.4%
	Induction of labor	5	16.1%
	Elective C/S	1	3.2%
	Emergency C/S	10	32.3%
Place of delivery		N (n = 33)	%
	Hospital	31	83.8%
	Home	2	16.2%
Pregnancy outcome due to RFM		N (n = 30)	%
	IUFD	16	53.3%
	Alive and well	7	23.3%
	Stillbirth	4	13.3%
	Infection	2	6.7%
	Jaundice	1	3.3%
Gestational age at delivery		N (n = 30)	%
	Less than 34 weeks	7	23.3%
	34 to 36 weeks	7	23.3%
	37-40 weeks	14	46.7%
	More than 40 weeks	2	6.7%
Five minutes APGAR score		N (n = 27)	%
	Less than 7	21	77.8%
	7 or more	6	22.2%
Birth weight in kilograms		N (n = 28)	%
	Less than 2.5 kg	12	42.9%
	2.5 to 3.5 kg	12	42.9%
	More than 3.5 kg	4	14.3%

## Discussion

This study represents the first of its kind to delve into the experiences of mothers who have encountered RFMs in the Red Sea state and Sudan, as far as we know. It offers valuable insights into the risk factors these women face, including the timing of RFMs and their possible link to stillbirth. While the findings suggest potential associations, the cross-sectional design and limited sample size prevent us from confirming these links definitively. Nonetheless, the study highlights important areas for further investigation.

The study reveals a diverse demographic breakdown of women presenting with RFM. It indicates that primigravida women are commonly associated with RFM, but a substantial number of multiparous women also experience this issue. Notably, approximately half of the participants (48.5%) fall within the 13-18 age group, with the majority (75.8%) having no formal education beyond literacy and none having pursued undergraduate studies. Lack of formal education may make it more difficult to gain knowledge about prenatal care and the importance of monitoring fetal movements, which could delay the onset of health-seeking behaviors. Low health literacy rates, which are linked to poorer pregnancy outcomes and may affect the frequency and perception of RFM, are also consistent with this demographic's limited educational background.

Moreover, early marriage, evident in 59.9% of participants marrying before 18, often correlates with early childbearing and potentially increases risks of complications during pregnancy, including RFM. The results highlight the need for tailored educational interventions that consider socio-cultural factors, aiming to improve maternal health literacy and awareness of prenatal risk indicators. Community-based programs focusing on adolescent and maternal health education could be pivotal in addressing both preventive measures for RFM and improving early responses to fetal health concerns.

Previous research in the same field that examined multiple risk factors for RFM failed to establish a clear association between educational level and the prevalence of RFM [26]. However, our findings highlight a concerning issue that demands further investigation, particularly regarding the significant proportion of illiterate women experiencing stillbirths. This observation cannot be overlooked and underscores the need for deeper exploration.

The antenatal period serves as a critical window for pregnant women to access interventions vital for their health and the well-being of their babies. Research indicates that receiving ANC at least four times during pregnancy enhances the likelihood of receiving essential maternal health interventions consistently throughout the antenatal period [27]. A study conducted in Bangladesh has demonstrated that ANC has led to a 46% reduction in the incidence of complications among high-risk pregnancies [28]. 

An important revelation from the data was that only a third of the study population had not encountered a professional healthcare worker during their gestation period (31%). This figure was notably lower than the findings of a study in Ethiopia, where 43.1% of women attended four or more antenatal visits during their pregnancy. The variance in results can be ascribed to the larger sample size in the latter study. Furthermore, the rate of adverse pregnancy outcomes among the group of women experiencing RFMs was alarmingly high at over 66%, a figure significantly surpassing that of another study conducted in the UK [29]. This disparity underscores the heightened rate of intrauterine fetal death among women experiencing RFMs, emphasizing the critical need to assess and address this issue within maternity units.

While this study focuses on a limited number of women presenting with the concern of RFMs and examines their pregnancy outcomes, the cross-sectional design of the study enabled the timely collection of data from women attending the largest tertiary referral hospital in the state. This approach ensured the inclusion of all women presenting with this issue and facilitated the gathering of pertinent data regarding their presentations. By comparing the baseline demographics with findings from other studies, the study sheds light on how pregnancy outcomes may be influenced by this antenatal complaint. Additionally, our study underscores the necessity for the development of both local and national guidelines concerning RFMs. Such guidelines are essential to standardize the evaluation and management practices for this condition, thereby reducing disparities in care [30].

Given that this is a preliminary observational study, the investigation and management strategies were left to the discretion of the attending clinician. It is important to note that there is currently no clear consensus on the optimal mode of assessment for RFMs, as various guidelines recommend different modes and timing of assessments [31].

Recommendations

Health authorities play a crucial role in improving maternal and fetal health outcomes by focusing on the development and implementation of standardized guidelines for the evaluation and management of RFMs. By prioritizing this area, they can ensure consistent and high-quality care for pregnant women, especially in regions with high rates of adverse pregnancy outcomes related to RFM. Allocating resources and funding toward initiatives aimed at enhancing ANC services such as conducting awareness campaigns to educate both the public and healthcare providers about the importance of early detection and management of RFM can help prevent adverse pregnancy outcomes. Health professionals are at the frontline of care for pregnant women experiencing RFM. It is crucial for them to stay updated on current guidelines and recommendations for assessing and managing RFM in pregnant women and collaborating with other healthcare providers and specialists to ensure comprehensive care for pregnant women experiencing RFM, and potential adverse outcomes is vital for holistic patient management.

Finally, encouraging pregnant women to attend regular ANC appointments, follow healthcare provider recommendations for a healthy pregnancy, and promptly report any changes in fetal movements and provide them with appropriate counseling and support can lead to timely interventions and improved outcomes in maternal and fetal well-being.

Study limitations

This study encountered several limitations and obstacles that hindered the research process. One significant challenge was the inability to access comprehensive information from the study subjects due to language barriers, which restricted the depth of data available. Additionally, data collection faced obstacles, including insufficient case numbers during the study period and the short post-delivery stay time of only two hours postoperatively for vaginal deliveries. Furthermore, a substantial portion of patient data was not documented in the notes, despite its critical relevance to the study, leading to incomplete records. Missing essential data such as Apgar scores, onset of labor, and fetal weight significantly impacted the analysis of key outcome measures. Moreover, the absence of established guidelines for the assessment and management of RFM meant that decisions regarding investigation and management were largely based on individual experience rather than standardized protocols. Moving forward, it is essential to enhance staff training in the realm of RFM. Emphasizing the recognition of the subjective maternal perception of RFM is crucial, rather than solely focusing on formal fetal counting methods, as has been the trend in recent years. This shift in emphasis can lead to a more comprehensive understanding of RFM and improve the quality of care provided to pregnant women experiencing this phenomenon.

## Conclusions

This study offers important insights into the demographic and clinical characteristics of women who present with RFMs. In addition to the substantial impact caused by social determinants including education level, early marriage, and limited access to prenatal care, a significant frequency of adverse pregnancy outcomes has been linked to RFM, underscoring the necessity of prompt and thorough treatment for women with this condition. Particularly in places where early marriage and low health literacy are common, the identification of modifiable risk factors, including health literacy and access to prenatal care, emphasizes the significance of customized interventions that give priority to maternal health education. The study also emphasizes the critical role of ANC in ensuring the health and well-being of pregnant women and their babies, along with the importance of the development of standardized local and national guidelines for RFM assessment and management in order to ensure equitable treatment and minimize disparities between maternal and neonatal outcomes. Further research is recommended to explore causal links between identified risk factors and RFM and to establish effective, evidence-based protocols that enhance clinical response and maternal education regarding fetal health monitoring.

## References

[1] Whitworth MK, Fisher M, Heazel A (2011). Reduced Fetal Movements. Green-top Guideline No. 57.

[2] Sergent F, Lefèvre A, Verspyck E, Marpeau L (2005). Decreased fetal movements in the third trimester: what to do?. Gynecol Obstet Fertil.

[3] Frøen JF, Heazell AE, Tveit JV, Saastad E, Fretts RC, Flenady V (2008). Fetal movement assessment. Semin Perinatol.

[4] Warrander LK, Batra G, Bernatavicius G (2012). Maternal perception of reduced fetal movements is associated with altered placental structure and function. PLoS One.

[5] Hussain AA, Yakoob MY, Imdad A, Bhutta ZA (2011). Elective induction for pregnancies at or beyond 41 weeks of gestation and its impact on stillbirths: a systematic review with meta-analysis. BMC Public Health.

[6] Pearson JF, Weaver JB (1976). Fetal activity and fetal wellbeing: an evaluation. Br Med J.

[7] Frøen JF, Arnestad M, Frey K, Vege A, Saugstad OD, Stray-Pedersen B (2001). Risk factors for sudden intrauterine unexplained death: epidemiologic characteristics of singleton cases in Oslo, Norway, 1986-1995. Am J Obstet Gynecol.

[8] Efkarpidis S, Alexopoulos E, Kean L, Liu D, Fay T (2004). Case-control study of factors associated with intrauterine fetal deaths. MedGenMed.

[9] Pagani G, D'Antonio F, Khalil A, Akolekar R, Papageorghiou A, Bhide A, Thilaganathan B (2014). Association between reduced fetal movements at term and abnormal uterine artery Doppler indices. Ultrasound Obstet Gynecol.

[10] Dutton PJ, Warrander LK, Roberts SA (2012). Predictors of poor perinatal outcome following maternal perception of reduced fetal movements-a prospective cohort study. PLoS One.

[11] Prefumo F, Sebire NJ, Thilaganathan B (2004). Decreased endovascular trophoblast invasion in first trimester pregnancies with high-resistance uterine artery Doppler indices. Hum Reprod.

[12] Nicolaides KH (2011). A model for a new pyramid of prenatal care based on the 11 to 13 weeks' assessment. Prenat Diagn.

[13] Herraiz I, Arbués J, Camaño I, Gómez-Montes E, Grañeras A, Galindo A (2009). Application of a first-trimester prediction model for pre-eclampsia based on uterine arteries and maternal history in high-risk pregnancies. Prenat Diagn.

[14] Melchiorre K, Leslie K, Prefumo F, Bhide A, Thilaganathan B (2009). First-trimester uterine artery Doppler indices in the prediction of small-for-gestational age pregnancy and intrauterine growth restriction. Ultrasound Obstet Gynecol.

[15] D'Antonio F, Rijo C, Thilaganathan B, Akolekar R, Khalil A, Papageourgiou A, Bhide A (2013). Association between first-trimester maternal serum pregnancy-associated plasma protein-A and obstetric complications. Prenat Diagn.

[16] Iacovella C, Franchi M, Egbor M, Bhide A, Thilaganathan B (2012). Relationship of first-trimester uterine artery Doppler to late stillbirth. Prenat Diagn.

[17] Myatt L, Clifton RG, Roberts JM (2012). The utility of uterine artery Doppler velocimetry in prediction of preeclampsia in a low-risk population. Obstet Gynecol.

[18] Singh T, Leslie K, Bhide A, D'Antonio F, Thilaganathan B (2012). Role of second-trimester uterine artery Doppler in assessing stillbirth risk. Obstet Gynecol.

[19] Frøen JF, Tveit JV, Saastad E (2008). Management of decreased fetal movements. Semin Perinatol.

[20] Mangesi L, Hofmeyr GJ, Smith V, Smyth RM (2015). Fetal movement counting for assessment of fetal wellbeing. Cochrane Database Syst Rev.

[21] Hofmeyr GJ, Novikova N (2012). Management of reported decreased fetal movements for improving pregnancy outcomes. Cochrane Database Syst Rev.

[22] Winje BA, Wojcieszek AM, Gonzalez-Angulo LY, Teoh Z, Norman J, Frøen JF, Flenady V (2016). Interventions to enhance maternal awareness of decreased fetal movement: a systematic review. BJOG.

[23] Carroll L, Gallagher L, Smith V (2019). Risk factors for reduced fetal movements in pregnancy: A systematic review and meta-analysis. Eur J Obstet Gynecol Reprod Biol.

[24] McCarthy CM, Meaney S, O'Donoghue K (2016). Perinatal outcomes of reduced fetal movements: a cohort study. BMC Pregnancy Childbirth.

[25] Bhatia M, Mitsi V, Court L (2019). The outcomes of pregnancies with reduced fetal movements: a retrospective cohort study. Acta Obstet Gynecol Scand.

[26] Smith V, Begley C, Devane D (2014). Detection and management of decreased fetal movements in Ireland: a national survey of midwives' and obstetricians' practices. Midwifery.

[27] Berhe B, Mardu F, Legese H (2019). Prevalence of anemia and associated factors among pregnant women in Adigrat General Hospital, Tigrai, northern Ethiopia, 2018. BMC Res Notes.

[28] Detlefs SE, Jochum MD, Salmanian B, McKinney JR, Aagaard KM (2022). The impact of response to iron therapy on maternal and neonatal outcomes among pregnant women with anemia. Am J Obstet Gynecol MFM.

[29] Wilton C (2013). Cork University Maternity Hospital Annual Report.

[30] (2024). Reduced Fetal Movements (Green-top Guideline No. 57). Green-top guideline No 57.

[31] Skornick RA (2004). Perinatal outcome among women with reduced perception of fetal movements. Am J Obstetrics Gynecol.

